# Charge collection kinetics on ferroelectric polymer surface using charge gradient microscopy

**DOI:** 10.1038/srep25087

**Published:** 2016-05-03

**Authors:** Yoon-Young Choi, Sheng Tong, Stephen Ducharme, Andreas Roelofs, Seungbum Hong

**Affiliations:** 1 Materials Science Division, Argonne National Laboratory, Lemont, IL 60439, USA; 2 Nanoscience and Technology Division, Argonne National Laboratory, Lemont, IL 60439, USA; 3 Department of Physics and Astronomy, Nebraska Center for Materials and Nanoscience, University of Nebraska, Lincoln, NE 68588, USA

## Abstract

A charge gradient microscopy (CGM) probe was used to collect surface screening charges on poly(vinylidene fluoride-trifluoroethylene) [P(VDF-TrFE)] thin films. These charges are naturally formed on unscreened ferroelectric domains in ambient condition. The CGM data were used to map the local electric current originating from the collected surface charges on the poled ferroelectric domains in the P(VDF-TrFE) thin films. Both the direction and amount of the collected current were controlled by changing the polarity and area of the poled domains. The endurance of charge collection by rubbing the CGM tip on the polymer film was limited to 20 scan cycles, after which the current reduced to almost zero. This degradation was attributed to the increase of the chemical bonding strength between the external screening charges and the polarization charges. Once this degradation mechanism is mitigated, the CGM technique can be applied to efficient energy harvesting devices using polymer ferroelectrics.

In ferroelectric materials, polarization charges are screened by surface charges in ambient condition because the screening charges are naturally attracted to unscreened polarization charges with opposite polarity on the surface^1^. Screening charges include injected charges, internal charges^2^ from defects or surface relaxation, and adsorbed charges from ambient such as hydroxyl ions (OH^−1^), hydrocarbons, and protons (H^+^)^3^,^4^,^5^. Control of the driving forces and kinetics of screening charges in ferroelectric materials is very important because they can induce a change of both polarization and surface potential. As such, many studies have focused on understanding the behavior of ferroelectric materials induced by the interactions between polarization charges and screening charges, which can influence the data storage density, signal reliability, and stability of ferroelectric memory devices^6^,^7^. Such interactions include anomalous polarization reversal^8^,^9^,^10^; local surface potential change^2^,^11^ due to the diffusion of screening charges to the grain boundaries; and intermittency, quasi-periodicity, and chaos in probe-induced ferroelectric domain switching^3^.

Recently, Hong *et al.* introduced charge gradient microscopy (CGM) imaging, which reveals the underlying polarization domain structure at high speed by scraping, collecting, and quantifying the surface screen charges^12^. The scraped charges, measured as current that scales with scraping rate, induces a charge gradient, which leads to the immediate relocation or refilling of the screen charges in the vicinity of the CGM probe. This technique is a reliable tool to study the complex dynamics of domain nucleation and growth induced by a biased tip in the absence of surface screening charges^12^.

Tong *et al.* further investigated the kinetics behind the mechanical removal of externally bonded screening charges and the kinetics of rescreening in ambient conditions using CGM and electrostatic force microscopy (EFM)^5^. They found that a minimum pressure needs to be applied to initiate mechanical removal of screening charges, and increasing the pressure leads to further removal of charges until a critical pressure is reached, when all screening charges are removed. Furthermore, the rescreening phenomena after the charge scraping with different pressures showed an exponential recovery with a single time constant, implying that the screening charge degree on ferroelectric surfaces can be controlled by mechanical means without affecting the polarization underneath^5^.

Another method of controlling the screening charges or charge flow by mechanical means is use of triboelectricity. Wang *et al.* invented a triboelectric nanogenerator that can convert mechanical energy into electricity with unprecedented efficiency^13^,^14^,^15^,^16^,^17^,^18^,^19^. It works by a coupling effect between triboelectrification and electrostatic induction through the contact separation or relative sliding between two materials that have opposite tribo-polarity. However, little is known about the mechanism by which screening charges are collected by CGM on the surface of polymer ferroelectric material, which is a promising candidate for mechanical energy harvesting^20^,^21^. For this paper, we investigated the mechanism behind the large current generation caused by local mechanical pressure using a CGM probe on poly(vinylidene fluoride-trifluoroethylene) [P(VDF-TrFE)] thin films. In addition, we investigated the mechanism behind the change of generated current as a function of the number of CGM scans across the sample.

## Results and Discussion

**Figure 1:**
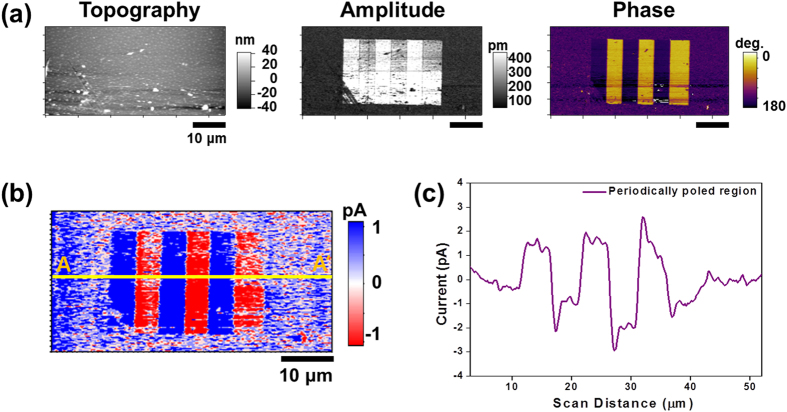
Topography, PFM, and CGM images along with a line profile of CGM contrast of periodically poled ferroelectric domains: (**a**) Topography, PFM amplitude, and PFM phase images of periodically poled regions. (**b**) CGM image on the same region as (**a**). (**c**) average line profile measured along A-A’ in (**b**).

To understand the mechanism of the current generation from the mechanically scraped charges from up and down domains on the surface of poly(vinylidene fluoride-trifluoroethylene) [P(VDF-TrFE)] thin films, which have remnant piezoresponse d_33_ value of −15 ± 4 pm/V and coercive voltage (V_c_) of 6.5 ± 0.3 V (Supplementary Fig. 1), we conducted CGM imaging on a periodically poled region with domain size of 5 μm × 20 μm. This region consisted of three negatively (−15 V to the tip) and three positively (+15 V to the tip) poled regions in an alternating manner. Before the poling process, the film consisted of mainly down domains, with possibly a small or even similar amount of up domains, as evidenced by the uniform piezoresponse force microscopy (PFM) phase and the low PFM amplitude outside the artificially poled regions in Fig. 1a 22. After the poling process, we conducted CGM imaging with loading force of 0.6 μN to the tip and scan frequency of 19.53 Hz over a scan size of 50 μm. Figure 1b clearly shows the domain contrasts in the CGM image, which correspond to the up (red) and down (blue) domains as verified by the PFM phase images (Fig. 1a). It is worth noting that P(VDF-TrFE) has a negative longitudinal piezoelectric coefficient^23^, which results in PFM phases of 0° and 180° for upward and down polarization vectors, respectively. We also measured the average peak current from the periodically poled region (Fig. 1c). The average value of the positive peak current is 1.57 ± 0.13 pA (collected charges: 3.65 ± 0.31 fC), and the negative peak current is −1.45 ± 0.59 pA (collected charges: −3.37 ± 1.37 fC). The average collected charges per unit area from the down and up domains are 0.81 ± 0.07 μC/cm^2^ and −0.75 ± 0.3 μC/cm^2^, respectively. We calculated the charges per unit area by dividing the collected charges over each domain by the diameter of the tip (90 nm) multiplied by the length (5 μm) of each domain^12^.

The shape of the CGM current signal over the up and down domains indicates that the CGM contrast in Fig. 1a originates from the charge flow from the ground to compensate for the overcharged CGM probe during the scraping of the charges^12^. This effect can be explained by the pileup of the external screening charges on the moving front of the probe and the charge refilling from ambient atmosphere at the trailing edge of the probe (Supplementary Fig. 2)^12^. In this case, the current flows over the domains, and its polarity does not depend upon the scan direction. The current is always positive over down domains, and negative over up domains during the CGM scans, which consist of trace and retrace scans as shown in Supplementary Fig. 3 12.

**Figure 2:**
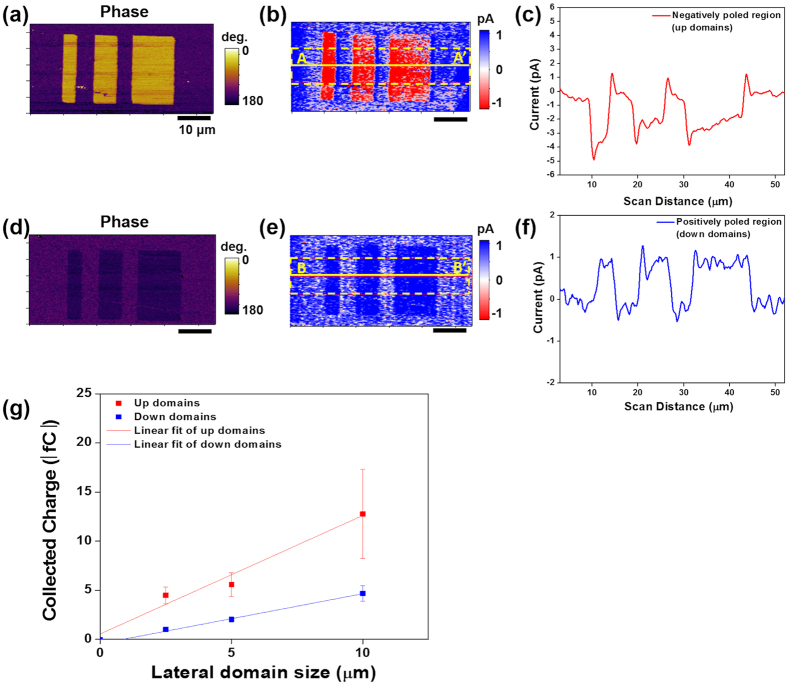
PFM phase and CGM images along with line profiles of CGM contrast of up and down poled ferroelectric domains: (**a**–**f**) PFM phase images, CGM images, and line profiles measured on (**a**–**c**) up domains and (**d**–**f**) down domains. CGM line profiles measured along A-A’ in (**b**) and B-B’ in (**e**). (**g**) Calculated collected charges from up and down domains as a function of poled width ranging from 2.5 to 10 μm.

We next explored whether the direction and amount of current from the up and down domains can be modulated. To that end, the domains in the same direction of either up or down were poled over regions of 2.5 μm × 20 μm, 5 μm × 20 μm and 10 μm × 20 μm. The results are displayed in Fig. 2. The average value of the negative current of collected from the up domains is −2.9 ± 0.83 pA. Also evident is a minor positive current peak at the domain boundaries above the domain current, which arises from the partial transfer of neighboring screening charges to the scraped region^12^. In the up domains, the collected charges changed from −4.5 ± 0.88 fC to −12.8 ± 4.5 fC as the length of poled region increased from 2.5 μm to 10 μm, as shown in Fig. 2g. The average collected charge per unit area is −1.55 ± 0.4 μC/cm^2^, as calculated from the linear fit of the up-domain data in Fig. 2g. In the down domains, the average value of positive current is 0.86 ± 0.014 pA, as calculated from Fig. 2f. In the down domains, the amount of collected charges increased from 1.0 ± 0.09 fC to 4.7 ± 0.8 fC as the length of poled region increased from 2.5 μm to 10 μm (Fig. 2g). The average collected charge per unit area is 0.47 ± 0.04 μC/cm^2^, as calculated from the linear fit of the down-domain data in Fig. 2g. Here, we confirmed that the direction and area of injected charges modulated both the direction and amount of current collection.

**Figure 3:**
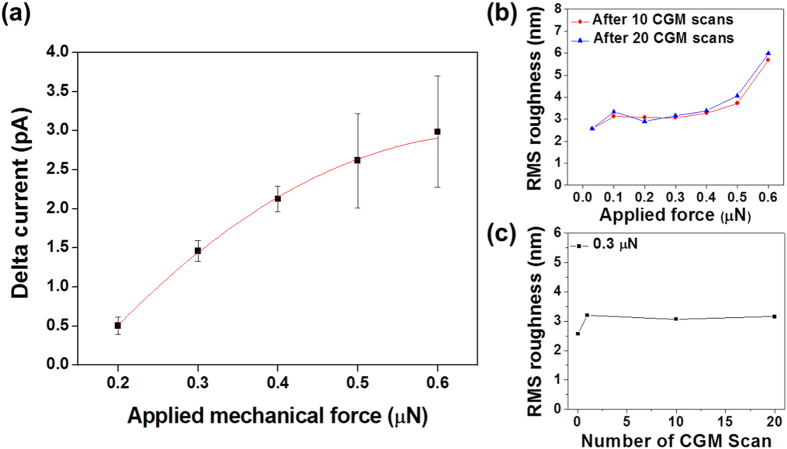
Delta current as a function of applied mechanical force and root mean square (rms) surface roughness as a function of applied force and number of CGM scans: (**a**) The difference between maximum and minimum current (i.e., delta current), which was measured on the periodically poled region in the line profiles, as a function of the mechanical force from 0.2 to 0.6 μN. (**b**) RMS surface roughness as a function of (**b**) applied force and (**c**) number of CGM scans under mechanical force of 0.3 μN measured by AFM.

To understand the effect of mechanical force on the CGM current, we measured the difference between the maximum and minimum current values (“delta current”) in periodically poled regions as a function of the mechanical force, ranging from 0.2 to 0.6 μN. As shown in Fig. 3a, the delta current increases with mechanical force in a nonlinear manner. This effect may be due to the plastic deformation of P(VDF-TrFE) with the increase of force. Even though a large amount of current is generated for mechanical force beyond 0.5 μN, the current was not sustainable with repeated scanning of the sample. In addition, the current becomes saturated beyond 0.5 μN, and its standard deviation increased probably due to the increase in the surface roughness (Fig. 3b). We did not apply a larger force than 0.6 μN due to a mechanical annealing effect, which can change nanoscale material properties and molecular orientation due to intensive local stress and can induce irreversible plastic deformation^22^. Therefore, we chose 0.3 μN as the optimum mechanical force for further study, which can generate sustainable current without significant change in the surface roughness for under 20 CGM scan cycles (Fig. 3c).

**Figure 4:**
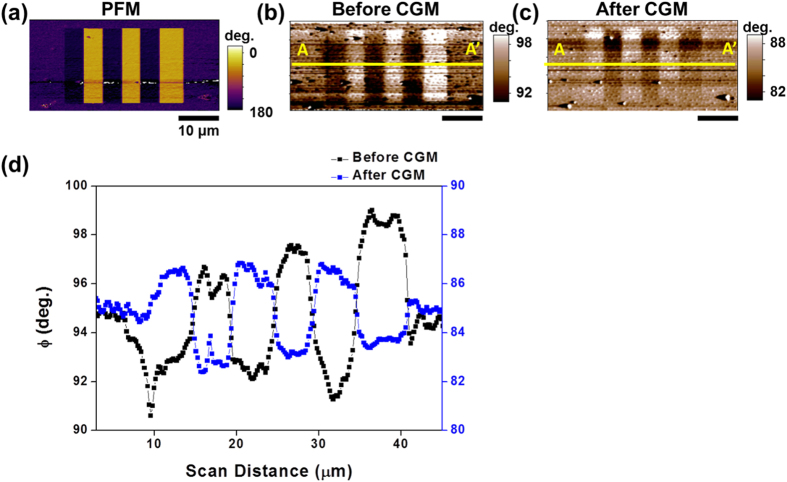
PFM, EFM images, and line profile of EFM phase before and after CGM scans: (**a**) PFM phase image measured on periodically poled region under mechanical force of 0.03 μN. (b, c) EFM phase image measured on periodically poled region (**b**) before and (**c**) after CGM scans. (**d**) Line profile of the EFM phase contrast before and after CGM scans with mechanical force of 0.3 μN along A-A’.

To reveal the mechanism of the current generation from periodically poled regions in Fig. 4a, we measured the surface potential of the poled domains using electrostatic force microscopy (EFM) before and after CGM imaging, as shown in Fig. 4b,c, respectively. Figure 4d shows the average line profile of the EFM contrasts in Fig. 4b,c along the scan direction.

Before CGM imaging, the variation of the surface potential on the periodically poled domains (Fig. 4b,d) was in agreement with the expected line profile of the up and down domains with injected screening charges on top of the domains^2^. On the other hand, after 19 CGM scans with applied force of 0.3 μN, the variation of the surface potential (Fig. 4c,d) matched the under-screened or unscreened up and down domains. In addition, a nearly symmetric variation of surface potential was observed after the CGM scans, as shown in Fig. 4d. The EFM images as functions of elapsed time and CGM scans in Supplementary Fig. 4 indicate that, after 2 CGM scans with loading force of 0.3 μN, the EFM phase contrast reduced in magnitude but maintained the color contrast (in the same charge polarity) compared with the EFM image for the periodically poled domains. This finding indicates that the over-screened charges coming from the charge injection can be scraped from the sample surface during the poling process. In addition, the EFM image changes its color contrast after 17 more scans, indicative of changing the surface state from slightly over-screened to under- or unscreened^2^.

**Figure 5:**
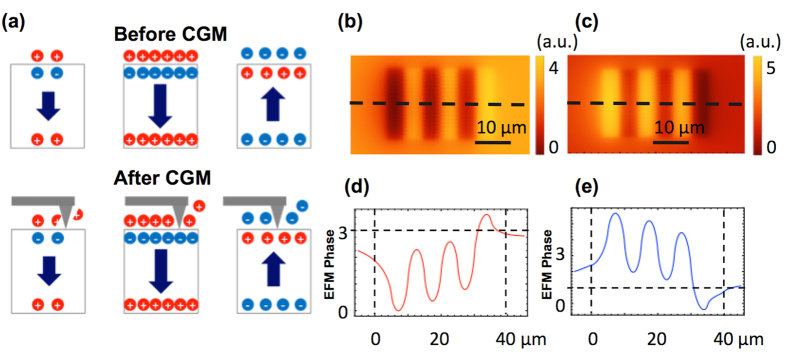
Schematic of hypothetical mechanism for screening charge distribution on up and down poled domains before and after CGM: (**a**) Cross-section view of screening and polarization charges in pristine state, followed by down and up poled domains (upper) before and (lower) after CGM scan. (**b**) Variation of expected surface screening charges before and after CGM scans on periodically poled ferroelectric domains.

The schematic of Fig. 5a shows our hypothetical mechanism, which can explain the nearly symmetric variation of the surface potential in periodically poled regions (Fig. 4) and the current generation over periodically poled domains (Fig. 1b,c). Based on the CGM, EFM, and PFM amplitude images in Figs 1b and 4c, and Supplementary Fig. 6, respectively, we propose that the amount of screening charges is larger on the down domains than the up domains, and the bonding strength of the external screening charges is stronger on the down domains than the up domains as well. These differences are consistent with other studies with ferroelectric materials. For example, Li *et al.* found that the difference of the adsorption energy of CH_3_OH and CO_2_ on BaTiO_3_ and Pb(Zr_0.52_Ti_0.48_)O_3_ on down and up domains is 3.4 kJ/mol and 2.4 kJ/mol, respectively^24^. Similarly, Garra *et al.* reported that the desorption energies of both water and 2-fluroethanol on down domains is 4 kJ/mol larger than those on up domains on periodically poled lithium niobate and BaTiO_3_ surfaces^25^,^26^. Therefore, the portion of scraped charges on the up domains is expected to be larger than that on the down domains, even though the magnitude of polarization of the up domains is smaller than that of the down domains. Figure 5b,c show the expected EFM phase images, based on our hypothetical mechanism and electrostatic potential calculations described in the Methods section, before and after CGM scans on the up and down domains. The expected EFM images and the line profiles shown in Fig. 5d,e are in good qualitative agreement with Fig. 4b–d and consistent with the asymmetric collected charges from the up and down domains shown in Fig. 2g 27.

**Figure 6:**
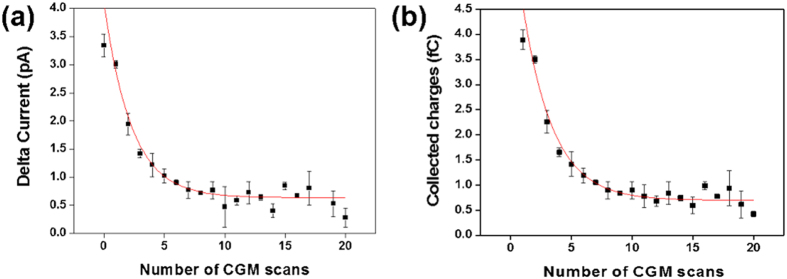
Delta currents and averaged collected charges measured over a single domain as a function of number of CGM scans with mechanical force of 0.3 μN: (**a**) Delta currents and (**b**) average collected charges over a single domain, which were measured in periodically poled region, as a function of number of CGM scans.

Figure 6 presents the collected charges as a function of number of CGM scans with mechanical force of 0.3 μN and scan frequency of 19.53 Hz. To confirm that the collected charges mainly came from the charge scraping, we also calculated and compared the expected charges generated by the direct piezoelectric effect and by scraping the screening charges. For the piezoelectric effect, we multiplied the converse piezoelectric coefficient, d_33_ of P(VDF-TrFE) (around -30 pC/N)^28^,^29^ by the applied mechanical force of 0.3 μN, which resulted in 0.009 fC. For the charge scraping, we multiplied the remanent polarization (10 μC/cm^2^)^30^ by the area of a hemisphere with a tip radius of 45 nm using the equation Q = σA = (P_r_ ⋅ n)A, where σ is surface charge density, P_r_ is a remnant polarization, n is the unit surface normal vector of A, and A is the area of contact^12^. Our calculation of Q = 0.64 fC indicates that most of the charge collection resulted from the charge scraping.

To investigate the repeatability of the current generation by CGM imaging, we measured the collected current and calculated the average collected charges over a single domain (see Fig. 6a,b) as a function of number of CGM scans. The collected current exponentially decayed with a time constant of about 14.81 seconds (~2.26 scans) and diminished when we increased the scan number beyond 20. Using the PFM amplitude, we confirmed that the underlying ferroelectric domains did not change after repeating the CGM scans over 30 times, as shown in Supplementary Fig. 7a. However, we were not able to collect any reliable CGM current after 20 CGM scans.

**Figure 7:**
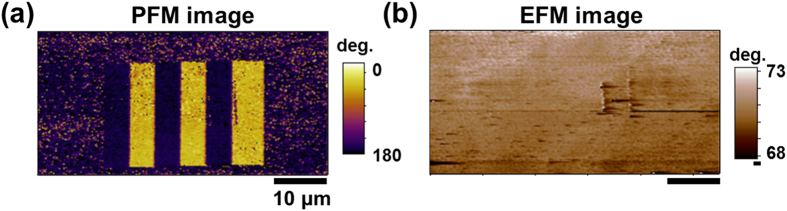
PFM and EFM images measured after 20 CGM scan cycles: (**a**) PFM phase and (**b**) EFM phase images after 20 CGM scan cycles.

To reveal the reasons behind the degradation of the CGM current after 20 CGM scans with mechanical force of 0.3 μN, we checked all possible mechanisms based on the finding that the up and down domains were not affected by the CGM scans in Fig. 7a and Supplementary Fig. 7a, whereas no significant EFM contrasts were observed in Fig. 7b. First, we considered the possible effect of debris transferred to the CGM tip from the sample surface, which could increase the contact resistance and decrease the CGM current. To check the effect of debris, we conducted another CGM scan with a new tip on the same region. The new tip did not collect any significant CGM current, as shown in Supplementary Fig. 7c, whereas the ferroelectric domains in the PFM mode could be imaged by using the new tip (see Supplementary Fig. 7b). This result excluded the possibility of debris transfer to the tip as the reason behind the degradation of the CGM contrast. It also excluded the possibility of increase in the recovery time. Second, we checked the mechanism where the sample surface could have been completely screened by internal screening charges, in which case the CGM tip would not collect any current^5^. Conducting-AFM (C-AFM) imaging with an applied voltage of 0.2 V to the tip on the same region confirmed that the region remained an insulator (see Supplementary Fig. 8). This condition would not be the case if the charge carriers were to freely move to the surface and buried interface between the P(VDF-TrFE) thin film and bottom electrode to internally screen the polarization. Third, we assumed that the bonding strength of the external screening charges to the surface increased significantly after repeated CGM scans. One of the possible mechanisms that could increase the bonding strength is the increase of surface roughness and entrapment of screening charges in the valleys of the surface^31^,^32^. However, as shown in Fig. 3b, the surface roughness did not change significantly after 20 cycles with a force below 0.4 μN. This finding excludes the possibility of the surface roughening causing the degradation of the CGM current.

The last mechanism we considered is the increase of the bonding strength of the external screening charges via a physical or chemical change of the surface. To test this hypothesis, we measured the adhesive force, which is pull-off force between the AFM tip and surface of P(VDF-TrFE) thin films before and after CGM scans with a mechanical force of 0.3 μN. The average adhesive force changed from 18.8 ± 3.4 nN to 66.6 ± 12.1 nN before and after CGM scans, respectively. This change indicates that the chemical bonding strength between the external screening charges and the polarization underneath increased significantly without any significant change in the surface roughness. We plan to address this effect in detail by investigating the mechanical, electrical, and chemical interaction at the interface.

## Conclusion

We have investigated the transduction of mechanical motion of the nanoscale CGM tip into electric current from the poled ferroelectric domains in P(VDF-TrFE) thin films. The current signals of P(VDF-TrFE) thin films dominantly originated from the ferroelectric domains when the surface screening charges were scraped with the CGM tip. In addition, we observed that the direction and area of injected charges modulated both direction and amount of current generation. The symmetric current generation in periodically poled regions was attributed to the different bond strengths between external charges and polarization charges. The lifetime of mechanical charge scraping on the polymer film was limited to 20 scan cycles, after which the current reduced to almost zero. This degradation was attributed to the increase of the chemical bonding strength between the external screening charges and the polarization charges due to change of the chemical properties of the film surface after the CGM scans. Our findings present a new challenge for the reliability of mechanical charge scraping, which is relevant to energy harvesting by charge scraping from polymer ferroelectrics.

## Methods

### Materials preparation

A 50-nm-thick P(VDF-TrFE) (75/25 mol%, MSI Sensors Inc.) film was deposited onto an Au/Cr/SiO_2_/Si substrate using a spin-coating process at rotating speed of 1500 rpm for 10 s. The film was subsequently annealed at 130 °C for 1 h on a hot plate. The structural characteristics of the film has been reported by Choi *et al.*^22^, where the pristine film contained only β phase with (200)/(110) planes oriented parallel to substrate based on the Grazing Incidence Wide-angle X-ray scattering (GIWAXS) images.

### Preparation of poled regions and PFM imaging

To make periodically poled regions with domain size of 5 × 20 μm, DC bias voltages of −15 V and +15 V were applied to the AFM tip. To make both up and down domains with domain size of 2.5 × 20 μm, 5 × 20 μm and 10 × 20 μm, the same bias voltages were applied to the AFM tip. Vertical PFM images were obtained near the contact resonance frequency of around 318.43 kHz with mechanical force of 0.03 μN using the vector PFM mode (Asylum research, MFP-3D). We also obtained Pt/Ir-coated silicon cantilevers (PPP-EFM, Asylum) with tip radius of 30 nm, Ti/Ir-coated silicon cantilevers (ASYELEC-02, Asylum), and Pt-wire tips (RMN 25Pt300B, Rocky Mountain Nanotechnology, LLC).

### Calibration of conducting AFM

To check the contact (Ohmic or Schottky) resistance, as well as the offset in voltage and current, we measured the conducting AFM using a standard sample of highly ordered pyrolytic graphite before the CGM measurement.

### CGM imaging

After the calibration, we performed CGM using grounded Pt-wire tips in electrically poled regions with applied mechanical forces of 0.2–0.6 μN and scan frequency of 19.53 Hz. Mechanical forces were determined by multiplying the inverse optical lever sensitivity (102.21 nm/V), the cantilever spring constant (5.26 N/m), and the cantilever deflection signal (0.186–1.116 V). During the CGM scans, we calibrated the offset voltage of around −83 mV present in the system.

### EFM imaging

Using the Ti/Ir coated tip, EFM images were obtained near the resonance frequency (312.95 and 319.04 kHz) with lift height of 50 nm and DC bias voltage of 3 V in the AC mode.

### Calculation of electrostatic potential and expected variation of EFM phase

We used a similar approach outlined by Kalinin *et al.*^10^ where the electrostatic potential was calculated using the tip height of 50 nm, effective thickness of adsorbates to be 2 nm, and polarization bound charges to be 4 μC/cm^2^, 8 μC/cm^2^ and 12 μC/cm^2^ on pristine surface, down domains and up domains. We also used the linear relationship between the surface potential and the EFM phase with a negative coefficient adjusted to fit the range of the change observed by the experiment. The absolute amount of scraped charges was the same for both down and up domains.

## Additional Information

**How to cite this article**: Choi, Y.-Y. *et al.* Charge collection kinetics on ferroelectric polymer surface using charge gradient microscopy. *Sci. Rep.* **6**, 25087; doi: 10.1038/srep25087 (2016).

## Supplementary Material

Supplementary Information

## References

[b1] Kalinin S. V. & Bonnell D. A. Nanoscale Phenomena in Ferroelectric Thin Films (ed Hong S. ) Ch. 8, 183–217 (Kluwer Academic Publishers, 2004).

[b2] Kim Y. *et al.* Origin of surface potential change during ferroelectric switching in epitaxial PbTiO_3_ thin films studied by scanning force microscopy. Appl. Phys. Lett. 94, 032907 (2009).

[b3] Ievlev A. V. *et al.* Intermittency, quasiperiodicity and chaos in probe-induced ferroelectric domain switching. Nat. Phys. 10, 59–66 (2014).

[b4] Zeng H. R., Yin Q. R., Li G. R., Luo H. S. & Xu Z. K. Abnormal piezoresponse behavior of Pb(Mg_1/3_Nb_2/3_)O_3_–30%PbTiO_3_ single crystal studied by high-vacuum scanning force microscope. J. Cryst. Growth 254, 432–436 (2003).

[b5] Tong S. *et al.* Mechanical removal and rescreening of local screening charges at ferroelectric surfaces. Phys. Rev. Applied 3, 014003 (2015).

[b6] Hong S. & Kim Y. Emerging Non-Volatile Memories (eds Hong S. *et al.* ) Ch. 7, 259–273 (Springer, 2014).

[b7] Kholkin A. L., Bdikin I. K., Shvartsman V. V. & Pertsev N. A. Anomalous polarization inversion in ferroelectrics via scanning force microscopy. Nanotechnology 18, 095502 (2007).

[b8] Bühlmann S., Colla E. & Muralt P. Polarization reversal due to charge injection in ferroelectric films. Phys. Rev. B 72, 214120 (2005).

[b9] Kim Y., Bühlmann S., Hong S., Kim S.-H. & No K. Injection charge assisted polarization reversal in ferroelectric thin films. Appl. Phys. Lett. 90, 072910 (2007).

[b10] Kim Y. *et al.* Screen charge transfer by grounded tip on ferroelectric surfaces. Phys. Status Solidi – R 2, 74–76 (2008).

[b11] Kalinin S. V. & Bonnell D. A. Local potential and polarization screening on ferroelectric surfaces. Phys. Rev. B 63, 125411 (2001).

[b12] Hong S. *et al.* Charge gradient microscopy. Proc. Nat’l. Acad. Sci. USA 111, 6566–6569 (2014). 24760831 10.1073/pnas.1324178111 PMC4020115

[b13] Wang Z. Triboelectric Nanogenerators as New Energy Technology for Self-Powered Systems and as Active Mechanical and Chemical Sensors. ACS Nano 7, 9533–9557 (2013). 24079963 10.1021/nn404614z

[b14] Fan F.-R., Tian Z.-Q. & Lin Wang Z. Flexible triboelectric generator. Nano Energy 1, 328–334 (2012).

[b15] Fan F. R. *et al.* Transparent triboelectric nanogenerators and self-powered pressure sensors based on micropatterned plastic films. Nano Lett. 12, 3109–3114 (2012). 22577731 10.1021/nl300988z

[b16] Zhu G. *et al.* Triboelectric-generator-driven pulse electrodeposition for micropatterning. Nano Lett. 12, 4960–4965 (2012). 22889363 10.1021/nl302560k

[b17] Wang S., Lin L. & Wang Z. L. Nanoscale triboelectric-effect-enabled energy conversion for sustainably powering portable electronics. Nano Lett. 12, 6339–6346 (2012). 23130843 10.1021/nl303573d

[b18] Bai P. *et al.* Integrated multilayered triboelectric nanogenerator for harvesting biomechanical energy from human motions. ACS Nano 7, 3713–3719 (2013). 23484470 10.1021/nn4007708

[b19] Zhu G. *et al.* Toward large-scale energy harvesting by a nanoparticle-enhanced triboelectric nanogenerator. Nano Lett. 13, 847–853 (2013). 23360548 10.1021/nl4001053

[b20] Kim D. *et al.* A spring-type piezoelectric energy harvester. RSC Advances 3, 3194 (2013).

[b21] Li D. J. *et al.* Polymer piezoelectric energy harvesters for low wind speed. Appl. Phys. Lett. 104, 012902 (2014).

[b22] Choi. Y.–Y. *et al.* Enhancement of local piezoresponse in polymer ferroelectrics via nanoscale control of microstructure. ACS Nano 9, 1809–1819 (2015). 25646972 10.1021/nn5067232

[b23] Bystrov V. S., Paramonova E. V., Bdikin I. K., Bystrova A. V., Pullar R. C. & Kholkin A. L. Molecular modeling of the piezoelectric effect in the ferroelectric polymer poly (vinylidene fluoride) (PVDF). J. Mol. Model. 19, 3591–3602 (2013). 23729009 10.1007/s00894-013-1891-z

[b24] Li D. *et al.* Direct *in situ* determination of the polarization dependence of physisorption on ferroelectric surfaces. Nat. Mater. 7, 473–477 (2008). 18469819 10.1038/nmat2198

[b25] Garra J., Vohs J. M. & Bonnell D. A. The effect of ferroelectric polarization on the interaction of water and methanol with the surface of LiNbO_3_(0001). Surf. Sci. 603, 1106–1114 (2009).

[b26] Zhao M. H., Bonnell D. A. & Vohs J. M. Influence of ferroelectric polarization on the energetics of the reaction of 2-fluoroethanol on BaTiO_3_. Surf. Sci. 603, 284–290 (2009).

[b27] Ievlev A. V., Morozovska A. N., Shur V. Y. & Kalinin S. V. Ferroelectric switching by the grounded scanning probe microscopy tip. Phys. Rev. B 91, 214109 (2015).

[b28] Bystrov V. S. *et al.* Nanoscale polarization patterning of ferroelectric Langmuir–Blodgett P(VDF-TrFE) films. J. Phys. D: Appl. Phys. 40, 4571–4577 (2007).

[b29] Vacche S. D. *et al.* The effect of processing conditions on the morphology, thermomechanical, dielectric, and piezoelectric properties of P(VDF-TrFE)/BaTiO_3_ composites. J. Mater. Sci. 47, 4763–4774 (2012).

[b30] Nguyen C. A., Mhaisalkar S. G., Ma J. & Lee P. S. Enhanced organic ferroelectric field effect transistor characteristics with strained poly(vinylidene fluoride-trifluoroethylene) dielectric. Org. Electron. 9, 1087–1092 (2008).

[b31] Naber R. C. G., Mulder M., de Boer B., Blom P. W. M. & de Leeuw D. M. High charge density and mobility in poly(3-hexylthiophene) using a polarizable gate dielectric. Org. Electron. 7, 132–136 (2006).

[b32] Narayanan Unni K. N., Bettignies R. D., Dabos-Seignon S. & Nunzi J.-M. A nonvolatile memory element based on an organic field-effect transistor. Appl. Phys. Lett. 85, 1823 (2004).

