# Supplementary material for: Charge collection kinetics on ferroelectric polymer surface using charge gradient microscopy
Source: Sci Rep. 2016 May 3;6:25087. doi: 10.1038/srep25087 (PMC4853730; doi:10.1038/srep25087)
Supplement: Supplementary Information [file srep25087-s1.doc]

**Charge collection kinetics on ferroelectric polymer surface**

**using charge gradient microscopy**

Yoon-Young Choi^1^, Sheng Tong^2^, Stephen Ducharme^3^, Andreas Roelofs^2^ and

Seungbum Hong1[[1]](#footnote-2)

^1^Materials Science Division, Argonne National Laboratory, Lemont, IL 60439, USA

^2^Nanoscience and Technology Division, Argonne National Laboratory, Lemont, IL 60439, USA

^3^Department of Physics and Astronomy, Nebraska Center for Materials and Nanoscience, University of Nebraska, Lincoln, NE 68588, USA

**1. Piezoresponse hysteresis loop of P(VDF-TrFE) thin films**

**
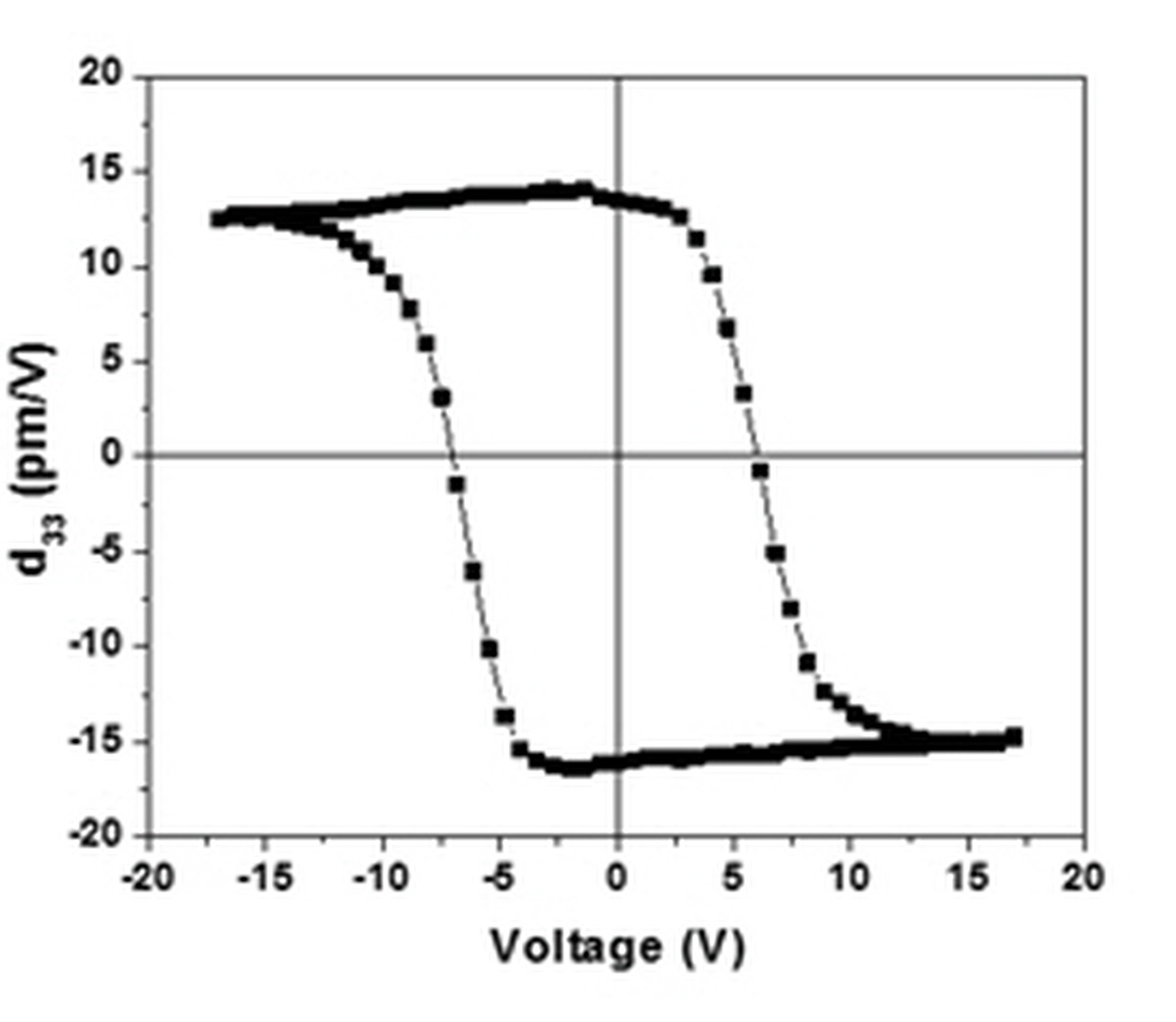
**

**Figure S1.** Piezoresponse hysteresis loop of 50 nm thick P(VDF-TrFE) thin films.

We fabricated a 50 nm thick P(VDF-TrFE) film by spin-coating and thermal annealing process, and confirmed that the film is composed of only β phase with (200)/(110) planes oriented parallel to substrate using grazing incidence wide-angle x-ray scattering **(**GIWAXS) imaging^1^. In addition, P(VDF-TrFE) film shows piezoresponse d_33_ value of -15 ± 4 pm/V and coercive voltage (V_c_) of 6.5 ± 0.3 V which is comparable with the macroscopic values of d_33_^2-3^. The piezoresponse hysteresis loops of P(VDF-TrFE) films were measured by dual ac resonance tracking PFM (DART-PFM) mode and at three different arbitrary points for four times at each position.

**2. Mechanism for CGM contrast**

**
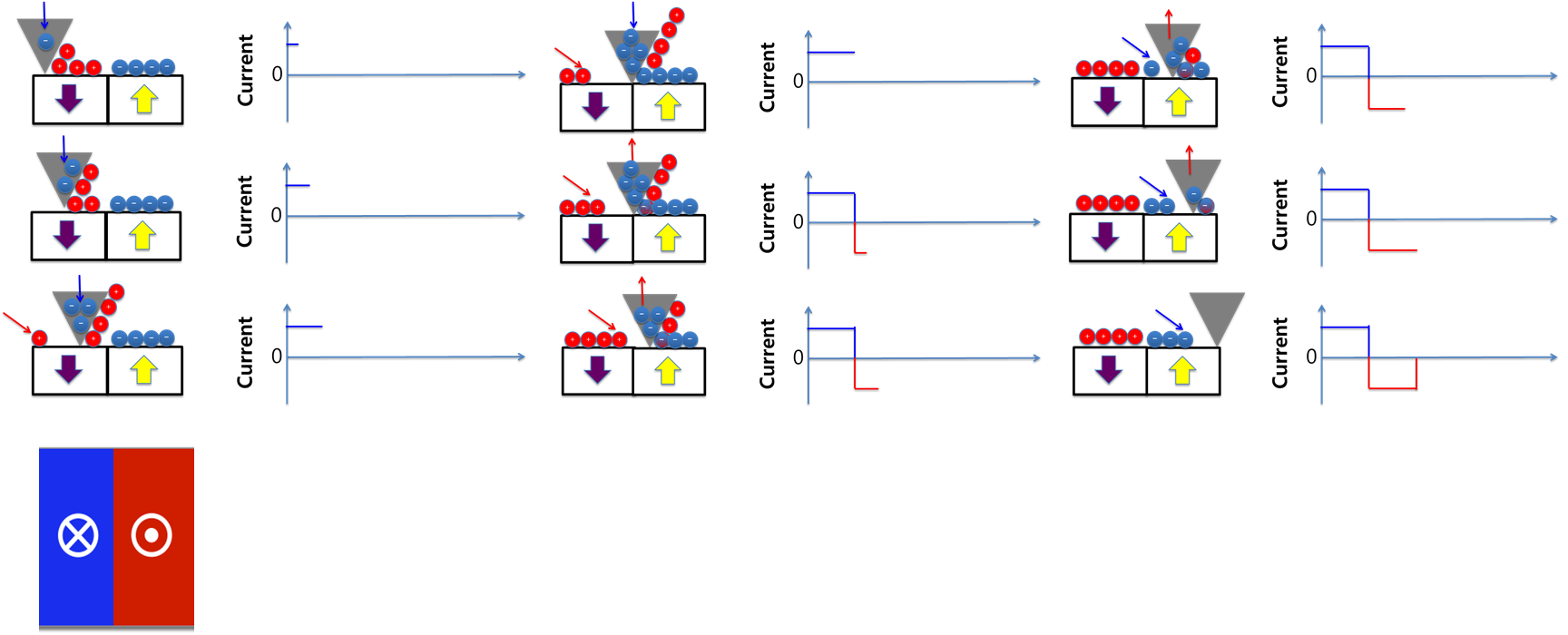
**

**Figure S2.** Schematic illustration of CGM mechanism based on the scraping of the external screening charges on the moving front of the CGM tip and replenishing of the screen charges from the ambient at the trailing edge of the tip with a delay. Reproduced with permission from Proc. Nat’l. Acad. Sci. USA [4]. Copyright 2014, National Academy of Sciences.

Hong *et al*. proposed four possible mechanisms for CGM contrasts based on the nature of screening and the way that the screening charges compensate the unscreened surface by the CGM tip^4^. Our research supports the mechanism that assumes continuous pileup of external screening charges on the moving front of the CGM tip, which eventually recombine with the screening charges of opposite polarity and instantaneous refill the screening charges on the trailing end of the CGM tip (Fig. S2). This mechanism works in the presence of the external screening charges, which are scraped by moving the tip, leading to the flow of the charges of opposite polarity from the CGM tip to the existing screening charges due to the change of electric potential of the tip. The mechanism also assumes that the trailing edge of the tip will leave uncompensated polarization charges that will be screened by the incoming screening charges from the ambient atmosphere over a period of time (from seconds to minutes depending on the ambient conditions)^5-9^. Furthermore, when the tip moves across the domain boundary (e.g., from down to up domains), the accumulated charges from the surface of the down domains at the moving front of the tip will recombine with the screening charges of opposite polarity, which reside on the up domains^4^.

**3. Trace and Retrace CGM images**


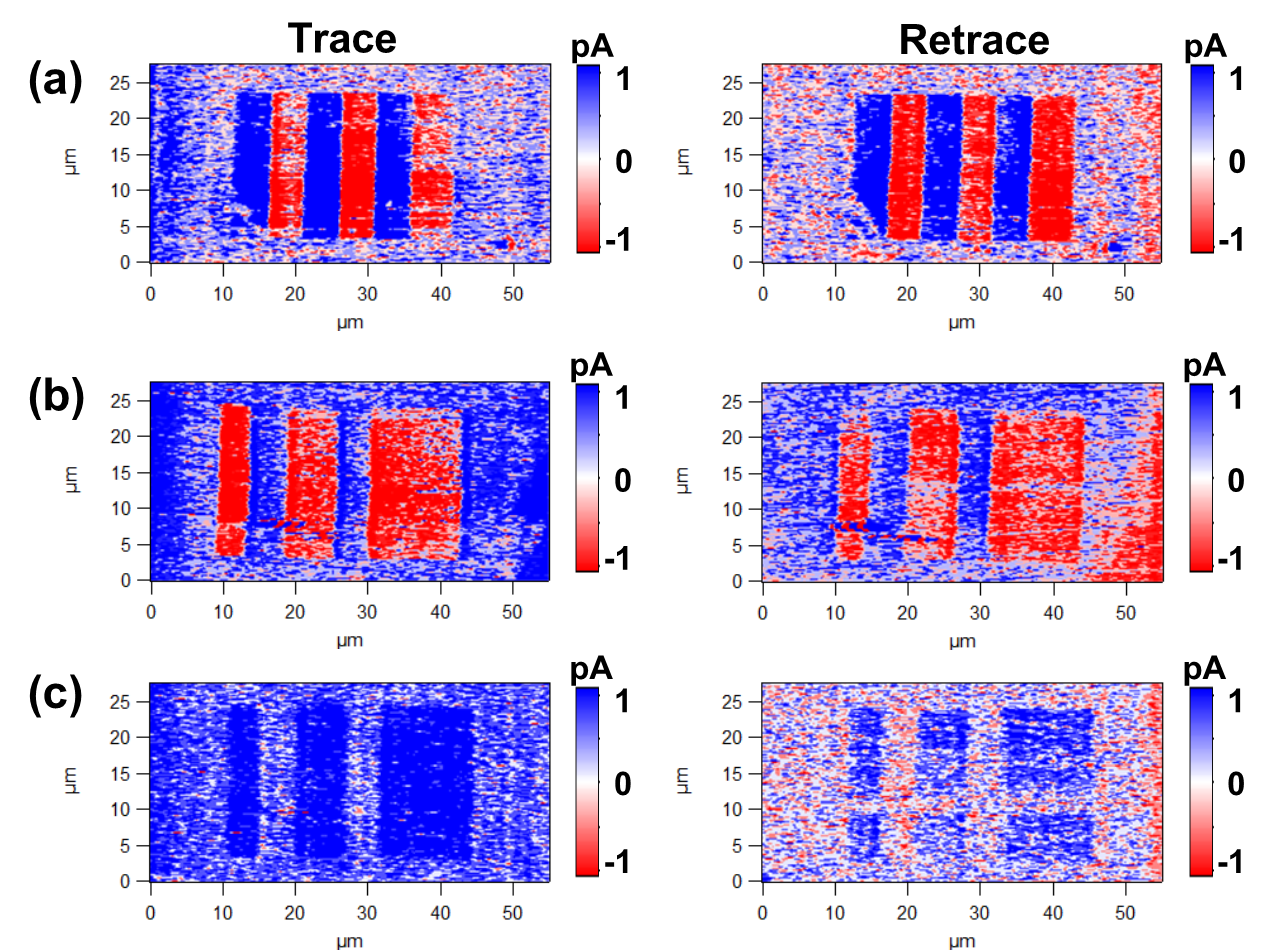


**Figure S3.** CGM images of both trace and retrace on (a) periodically poled region, (b) up domains, and (c) down domains.

Fig. S3 show the CGM images acquired at scan frequency of 19.53 Hz from both trace and retrace scans over periodically poled (Fig. S3(a)), up poled (Fig. 3(b)), and down poled (Fig. S3(c)) ferroelectric domains on P(VDF-TrFE) films with mechanical force of 0.6 µN. Both trace and retrace CGM images show the same contrast for the poled regions. CGM contrasts on the pristine region show noisy features with random variation depending on the direction of CGM scan, indicative of a weak dominance of down domains in the pristine region. Based on the observation on the poled regions, the negative current always comes from the down domains, whereas the positive current comes from the up domains during the CGM scans with little contribution from the domain boundary.

**4. Evolution of EFM Contrasts as a Function of CGM Scans and Elapsed Time**


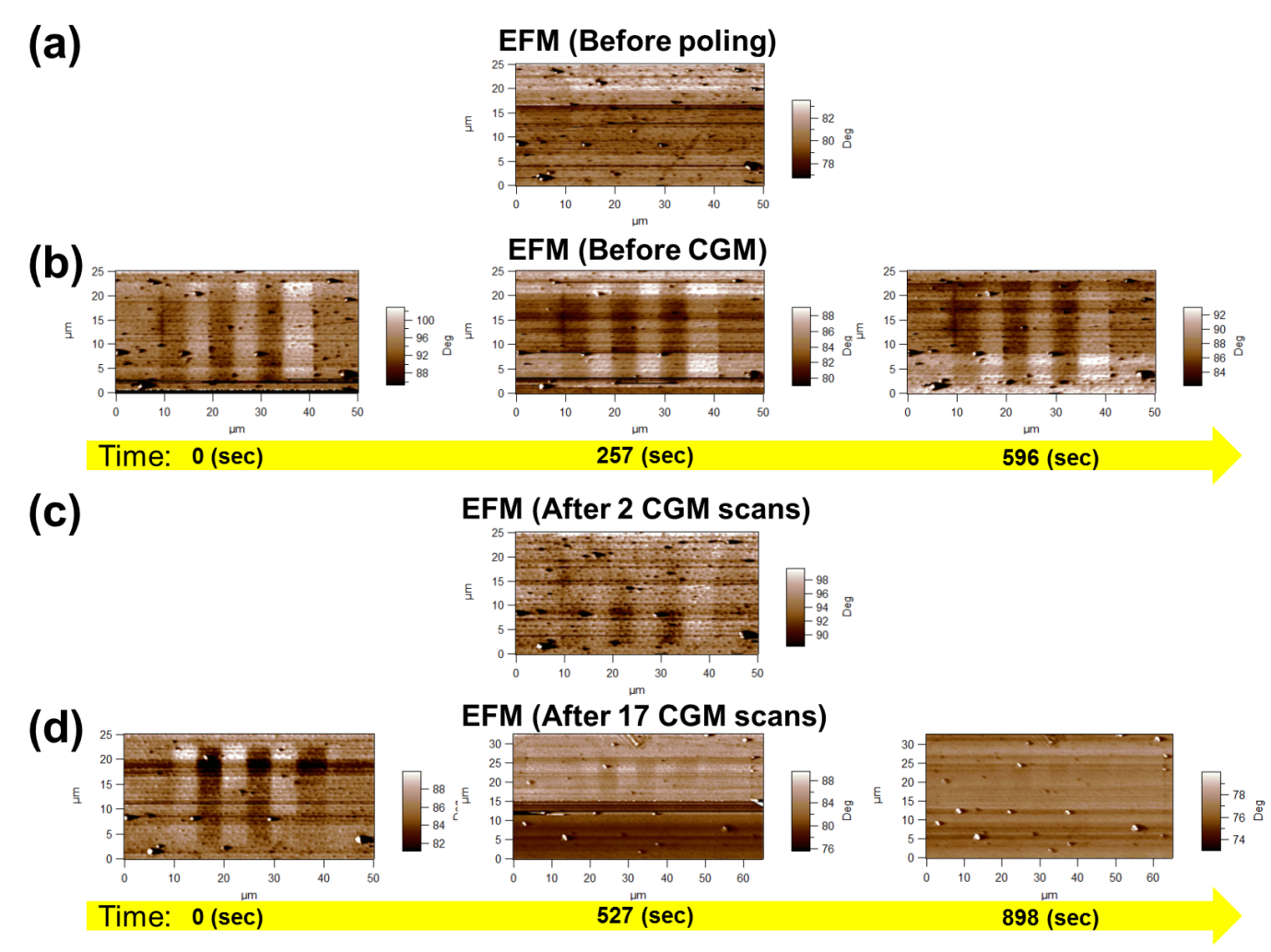


**Figure S4.** EFM images (a) before poling, (b) after poling and before CGM scans as a function of time (from left to right), (c) after 2 CGM scans with mechanical force of 0.3 μN, and (d) after 17 CGM scans with mechanical force of 0.3 μN as a function of time (from left to right). The yellow arrows below EFM images in (b) and (d) indicate the elapsed time after the first EFM image.

To observe the evolution of the surface potential distribution over periodically poled ferroelectric domains as a function of CGM scans and elapsed time, we conducted a series of experiments using both EFM and CGM. First, we imaged the pristine region using EFM as shown in Fig. S4(a), which indicates uniform EFM phase contrast. Second, we formed three down and up domains by the poling process, and obtained three consecutive EFM phase images, as shown in Fig. S4(b). All EFM phase images (elapsed time: 0, 257, and 596 seconds) after the poling process showed similar contrasts of alternating dark and bright regions for the down and up domains. Third, we acquired EFM phase image after 2 CGM scans (Fig. S4(c)) and found that the EFM phase contrast reduced in magnitude but maintained the color contrast (in the same charge polarity) compared with the EFM image of the periodically poled domains in Fig. S4(b). Lastly, we imaged the periodically poled domains after 17 CGM scans using EFM (Fig. S4(d)), and clearly observed the inversion of the EFM phase contrast when compared with Figs. S4(b) or S4(c), indicative of the transition from the over-screened state to either under- or unscreened. The evolution of EFM phase images in Fig. S4(d) shows that the under- or unscreened state is not sustainable and changes to fully screened state, as evidenced by the negligible EFM phase contrast after 898 seconds.

**5. Recovery of screening charges**

| 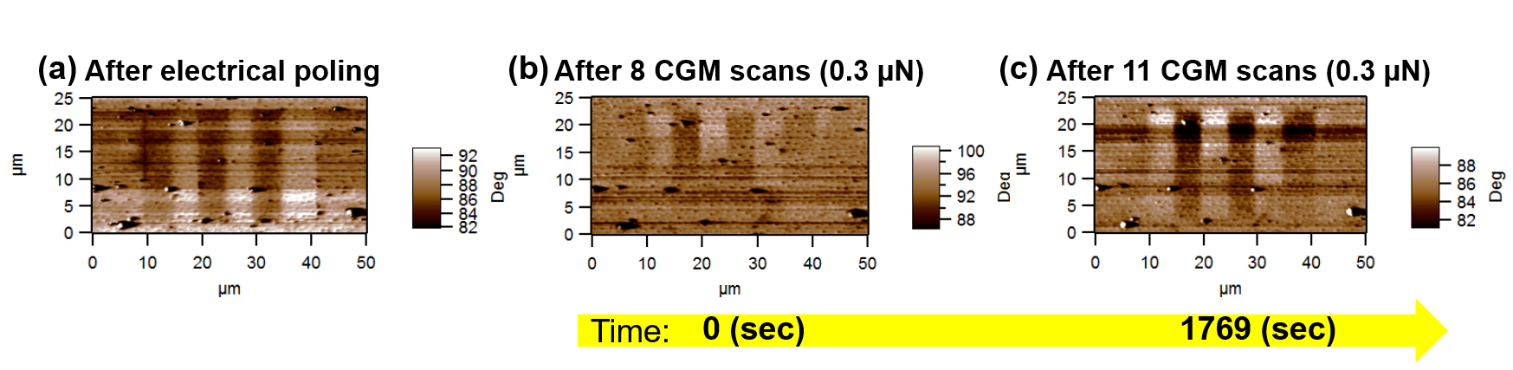 |
| --- |
| Figure S5. EFM phase images measured (a) after electrical poling, (b) after 8 CGM scans with mechanical force of 0.3 μN and (c) both after 11 CGM scans with mechanical force of 0.3 μN and after 1769 seconds from (b). |

We tested the hypothesis of the increase in the screening charge recovery time in the following manner.

1. We checked the screening charge recovery time based on the EFM phase images after a certain number of CGM scans followed by a given elapsed time.

2. Fully recovered state after CGM was characterized by no significant EFM contrast (e.g. Fig. S4(d) or Fig. 7(b)) whereas initially poled (slightly over-screened) or under-screened state was characterized by periodic high and low contrasts as shown in Figs. 4(b) and (c).

3. Estimated recovery time for the surface after 17 CGM scans was less than 900 seconds: In Fig. S4d, we acquired the EFM phase images as a function of time after 17 CGM scans and after around 898 seconds EFM phase image contrast almost fully disappeared, indicative of a fully recovered (screened) state.

4. EFM images of the surface that underwent 8 CGM scans followed by 1,769 seconds of rest and 11 more CGM scans on the same region: We expect that the EFM images will undergo a change similar to that observed in Fig. 4(d) where initially poled state (Fig. S5(a)) changes to under-screened state (Fig. S5(b)) right after the 8^th^ CGM scan and then change to fully recovered state like the rightmost image in Fig. S4(d) where no significant EFM contrast is observed. Then, after another round of 11 CGM scans on the same region, we found that the fully recovered state changes back to under-screened state as evidenced in Fig. S5(c).

5. EFM images of the surface that underwent 20 CGM scans followed by one day of rest (> 24 hours): We found that the initially poled state directly converted to a fully recovered state without any hint of under-screened state found in both cases of #3 and #4 mentioned above. This fully recovered state prolonged for more than a day as shown in Figure 7(b).

Based on our tests, we believe the chemical nature of the surface is modified after mechanical scanning and the surface charges cannot be removed by CGM further, without applying more force that results in mechanically annealed surface reported by our group^1^.

**6.** **PFM Images before CGM Scans**

**
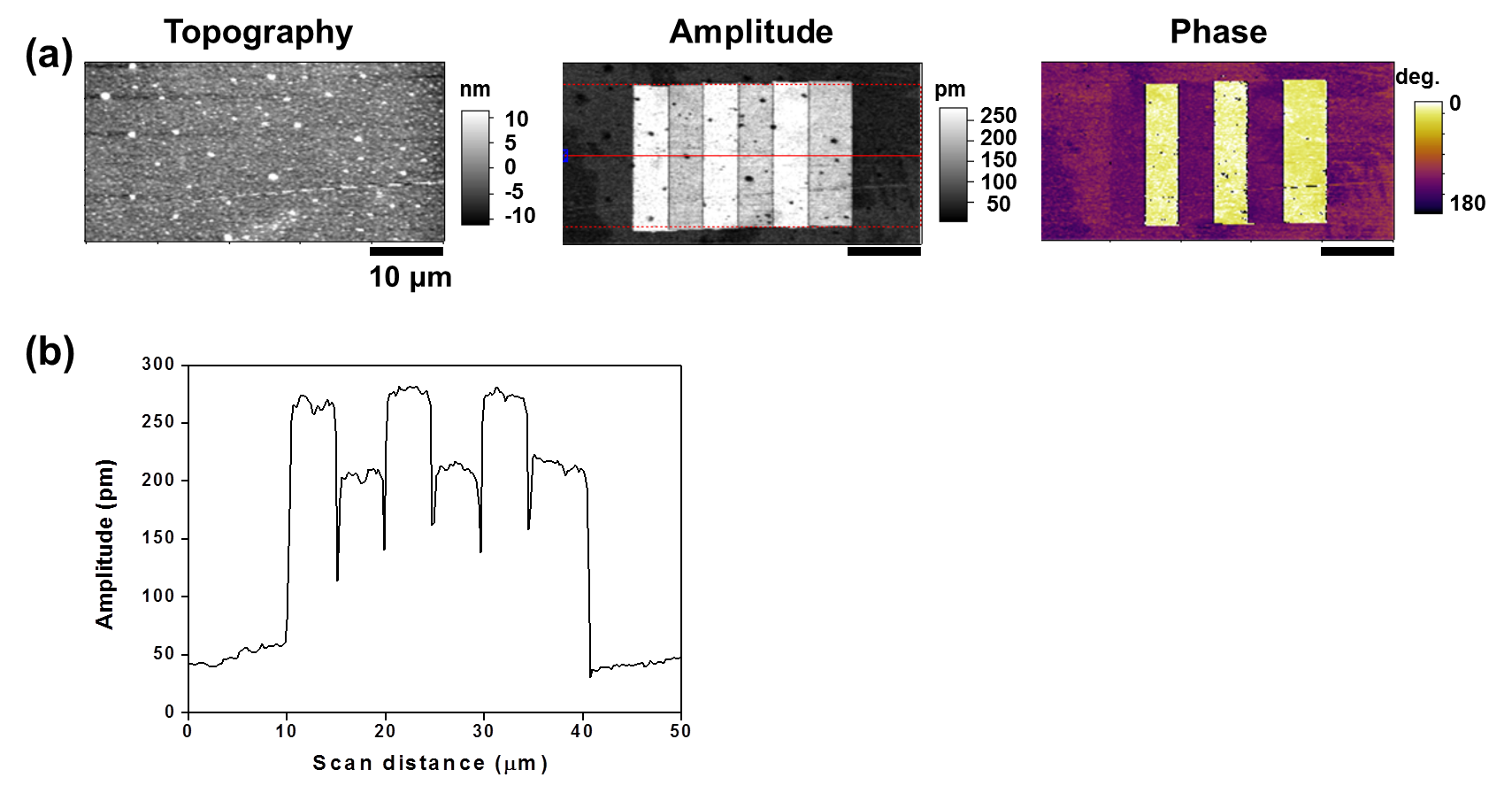
**

**Figure S6.** (a) Topography, PFM amplitude, and PFM phase images before CGM scans measured under mechanical force of 0.03 μN. (b) Line profile of PFM amplitude.

We applied DC bias voltages of -15 V and +15 V to the AFM tip to pattern periodically poled regions with 5 µm × 20 µm size of up and down domains. To confirm the polarization direction and magnitude of polarizations, we obtained topography, PFM amplitude, and PFM phase images on periodically poled regions with a mechanical force of 0.03 µN, which is in the range of normal contact force, before CGM scans.

Fig. S6(a) indicates the formation of up and down domains on periodically poled regions. Fig. S6(b) shows the line profile along the red line in the PFM amplitude image in Fig. S6(b). The line profile represents the variation between up and down domains, from which the PFM amplitudes are measured to be 269.9 ± 7.43 pm for down domains and 203.9 ± 6.18 pm for up domains. This difference indicates that the polarization of the down domains is larger than that of the up domains (also see Fig. 5(a) in main text).

**7.** **PFM and CGM Images obtained using a fresh CGM tip**


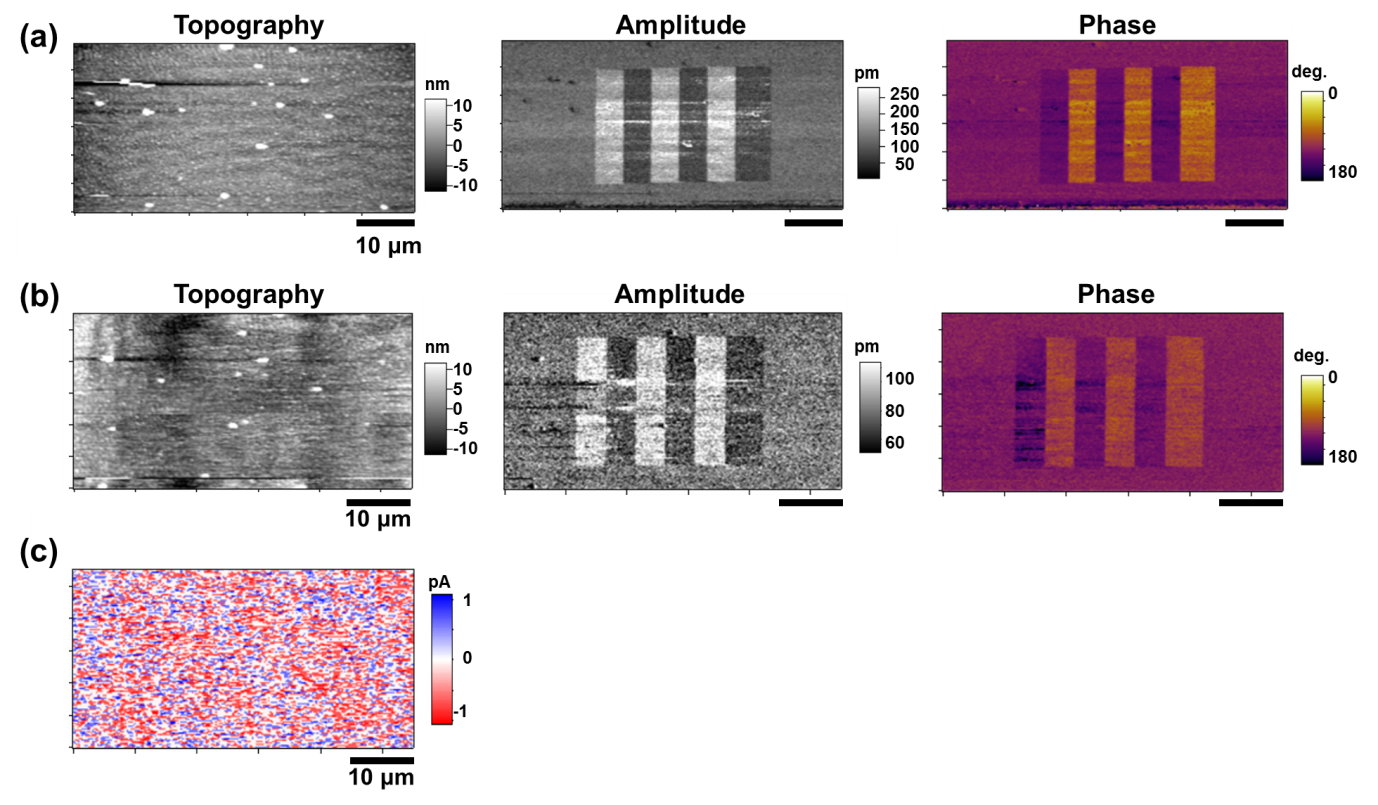


**Figure S7.** (a) Topography, PFM amplitude, and PFM phase images acquired under mechanical force of 0.03 µN after 30 CGM scans with mechanical force of 0.3 µN. (b) Topography, PFM amplitude, and PFM phase images and (c) CGM image obtained on the same region in (a) using a new Pt-wire tip.

As reported by Hong *et al*.^4^, debris can be transferred to the CGM tip from the sample surface, leading to an increase in the contact resistance and a decrease in the CGM current signal. To investigate the possible contamination of the CGM tip after repeated CGM scans, we conducted CGM imaging for 30 scans and confirmed no CGM contrast (not shown here). Fig. S7(a) shows the topography, PFM amplitude, and PFM phase images obtained with the same CGM tip. As discussed in the main text, current signals ceased after 20 CGM scans, we could still observe up and down domains using PFM.

Next, we replaced the used CGM tip with a new CGM tip and obtained PFM and CGM images for the new Pt-wire tip over the same region of Fig. S7(a). Again, the up and down domains were observed in the PFM image, but no CGM contrast, as shown in Fig. S7(c). These results suggest that the contamination of the CGM tip is not the main reason behind the degradation of the CGM contrast.

**8.** **Conducting-AFM Image after 30 CGM Scans**


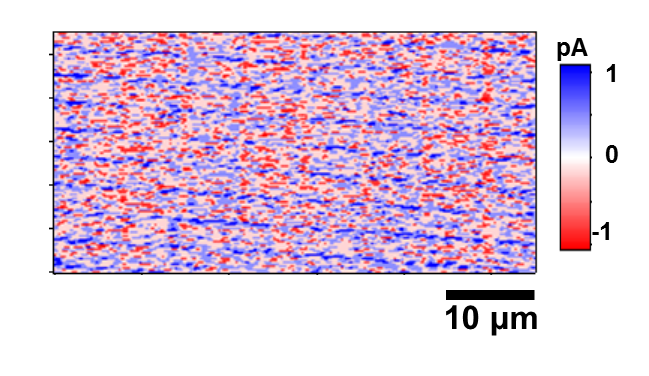


**Figure S8.** Conducting-AFM image of the same region (30 CGM scans) in Fig. S6 with a bias voltage of 0.2 V applied to the AFM tip.

Another possible mechanism for the degradation of the CGM contrast as a function of CGM scans is the internal screening as discussed by Hong *et al.*^4^ Charged defects such as vacancies or interstitials can create gap states within the band gap, as well as internal conduction paths either inside the film or close to the surface and the interface between the polymer ferroelectric film and the bottom electrode. More specifically, the reason for this internal screening could be the increase in the density of charged defects as we increase the CGM scans, and those defects will either move to both surface and interface to screen the polarization or they make the film leaky by creating delocalized gap states to enhance the film conductivity, in which case electrons and holes can screen the polarization. In either case, we claim that the film conductivity will increase because of the film thickness of 50 nm. If the charged defects move to the surface and the buried interface to screen the polarization, they will create conducting layers that will likely be connected in thickness direction as well due to the overlap of those layers.

As such, we acquired C-AFM image with a bias voltage of 0.2 V applied to the tip on the same region as that shown in Fig. S6 where we conducted 30 CGM scans. We found no meaningful current pattern in the C-AFM image and the current level was very low when compared with current level (> 20 pA) of conducting filaments in TiO_2_ films with similar thickness (40 nm) and the same bias voltage of 0.2 V to the tip^10^. There are some weak signals probably from scraped charges on the ferroelectric domains but no leakage current, which excludes the mechanism where the film is fully conductive and can supply screening charges freely to both the surface and the buried interface between P(VDF-TrFE) film and the bottom electrode.

**9.** CGM images obtained with mechanical forces of 0.5 μN and 0.6 μN

| 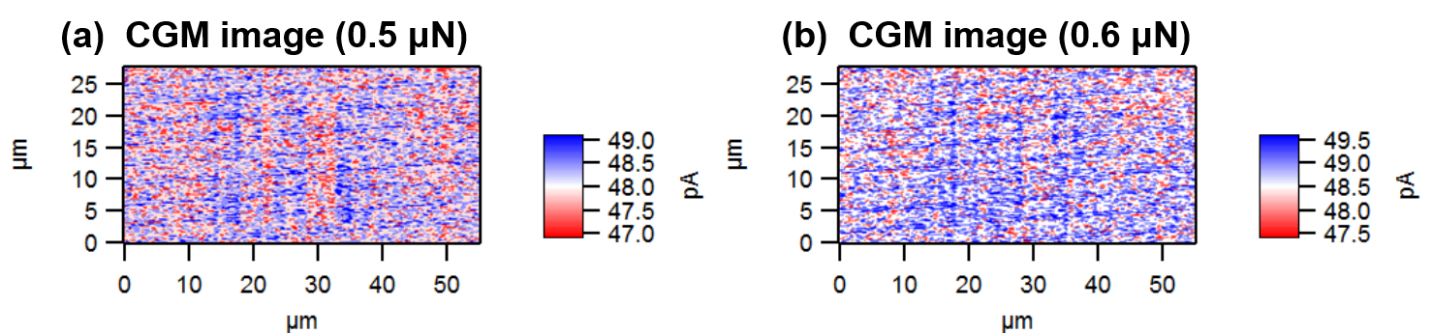 |
| --- |
| **Figure S9**. CGM images obtained with mechanical force of (a) 0.5 μN and (b) 0.6 μN in the same location where we conducted 20 CGM scans with mechanical force of0.3 μN. |

Our group reported that we could control the portion of screening charge being scraped by increasing the loading force^5^. However, the problem with the P(VDF-TrFE) thin films when compared with the oxide films or bulk samples is that we cannot apply high enough force due to the mechanical annealing effect^1^.

Regarding the reason behind no CGM contrast after 20 CGM scans, we think that the chemical bonding strength between the external screening ions and the polarization charges increased to the point that we cannot avoid the mechanical annealing effect. This is well illustrated in Fig. S9 where we attempted to increase the loading force up to 0.6 µN, where no significant contrast (or some weak contrast in Fig. S9(a)) can be observed.

**10. Point Spectroscopy of C-AFM current as a function of loading force**
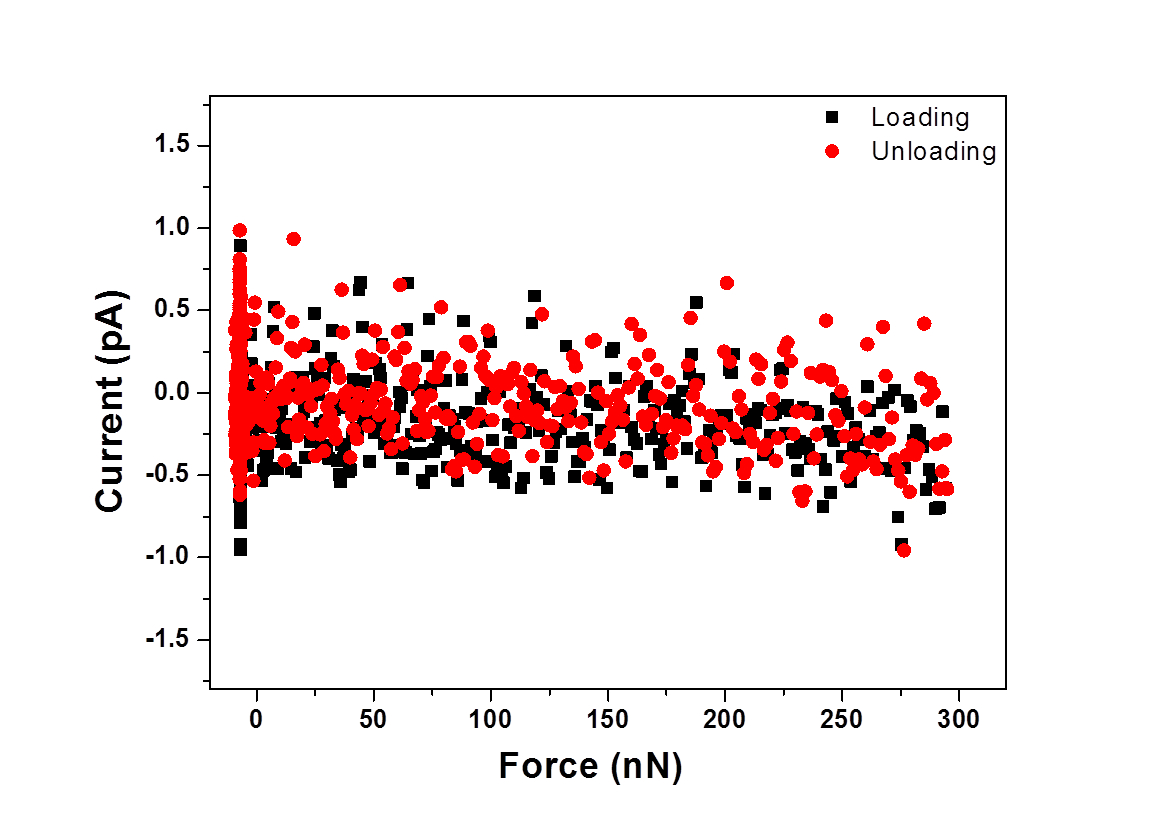


**Figure S10.** C-AFM measurement while applying compressive force of 0.3 µN to the film surface.

To confirm that the CGM contrast comes from the scraping of screening charges induced by both the loading force of 0.3 µN and the lateral motion of the CGM, we measured the C-AFM current signals as a function of the loading force at a fixed point (no scanning motion). Fig. S8 was obtained from the force-displacement and current-displacement curves measured simultaneously on the same point. The maximum force applied to the P(VDF-TrFE) thin films during this measurement was 0.3 µN.

As shown in Fig. S10, we found no meaningful current signals while applying compressive force of up to 0.3 µN, which excludes the possibility of pure piezoelectric charge generation and indicates that the charges are scraped from the scanning motion of the CGM tip under a mechanical force.

**References**

1. Choi. Y. –Y. *et al.* Enhancement of local piezoresponse in polymer ferroelectrics via nanoscale control of microstructure. *ACS Nano* **9**, 1809-1819 (2015).
2. Bune, A. V. *et al.* Temperature dependence of elastic, dielectric, and piezoelectric properties of “single crystalline’’ films of vinylidene fluoride trifluoroethylene copolymer Piezoelectric and pyroelectric properties of ferroelectric Langmuir–Blodgett polymer films. *J. Appl. Phys.* **85**, 7869 (1999)
3. Park, M. *et al.* The piezoresponse force microscopy investigation of self-polarization alignment in poly(vinylidene fluoride-co-trifluoroethylene) ultrathin films. *Soft Matter*, **8**, 1064 (2012)
4. Hong, S. *et al.* Charge gradient microscopy. *Proc. Nat’l. Acad. Sci. USA* **111**, 6566-6569 (2014).
5. Tong, S. *et al.* Mechanical removal and rescreening of local screening charges at ferroelectric surfaces. *Phys. Rev. Applied* **3**, 014003 (2015).
6. Kalinin, S. V. & Bonnell, D. A. Local potential and polarization screening on ferroelectric surfaces. *Phys. Rev. B* **63**, 125411 (2001).
7. Kalinin, S. V. & Bonnell, D. A. Screening phenomena on oxide surfaces and its implications for local electrostatic and transport measurements. *Nano Lett.* 4, 555 (2004).
8. Kim, Y. *et al*. Effect of local surface potential distribution on its relaxation in polycrystalline ferroelectric films. *J. Appl. Phys.* **107**, 054103 (2010).
9. Ievlev, A. V. *et al.* Intermittency, quasiperiodicity and chaos in probe-induced ferroelectric domain switching. *Nat. Phys*. **10**, 59 (2014).
10. Chang, S. H. *et al*. X-ray Irradiation Induced Reversible Resistance Change in Pt/TiO_2_/Pt Cells. *ACS Nano* **8**, 1584 – 1589 (2014).

1. Corresponding author: [hong@anl.gov](mailto:hong@anl.gov) [↑](#footnote-ref-2)
